# Adenylosuccinic Acid Is a Non-Toxic Small Molecule In Vitro and In Vivo

**DOI:** 10.3390/ph16101458

**Published:** 2023-10-13

**Authors:** Cara A. Timpani, Lorna Rasmussen, Emma Rybalka

**Affiliations:** 1 Institute for Health and Sport (IHeS), Victoria University, Melbourne, VIC 8001, Australia; cara.timpani@vu.edu.au; 2 Inherited and Acquired Myopathy Program, Australian Institute for Musculoskeletal Science (AIMSS), St Albans, VIC 3021, Australia; 3 Department of Medicine—Western Health, Melbourne Medical School, The University of Melbourne, St Albans, VIC 3021, Australia; 4 Cerberus Sciences, Scoresby, VIC 3179, Australia; lorna@cerberus.net.au; 5 Division of Neuropaediatrics and Developmental Medicine, University Children’s Hospital of Basel (UKBB), 4031 Basel, Switzerland

**Keywords:** adenylosuccinic acid, Duchenne muscular dystrophy, myopathy, metabolic disease, skeletal muscle, toxicology, drug development

## Abstract

Adenylosuccinic acid (ASA) is a small molecule dicarboxylate that could be a strong clinical development candidate for inherited myopathies involving dysregulated purine nucleotide metabolism. Currently, there are no published pharmacokinetic/dynamic or toxicology data available, although 10-year clinical trial data on Duchenne muscular dystrophy patients suggests it is a chronically safe drug. In this study, we tested the toxicity of ASA to cultured myoblasts in vitro and its acute systemic toxicity in mice. ASA is a non-toxic small molecule with an LD_50_ > 5000 mg/kg. Some background necrotic foci in the liver, kidney and gastrointestinal tract were shown that are likely incidental but warrant follow-up sub-/chronic oral exposure studies.

## 1. Introduction

Adenylosuccinic acid (ASA) is a small molecule metabolite that was clinically evaluated for Duchenne muscular dystrophy (DMD) in the 1980–1990s [1]. Dystrophin-deficient muscles manifest extensive metabolic anomalies [2,3] and ASA had previously been shown in vitro to correct aberrant mitochondrial isocitrate dehydrogenase activity [4]. Although the trial showed that ASA could attenuate the clinical biomarkers of DMD (e.g., serum creatine kinase) [1], the research program was ultimately abandoned after a decade due to a lack of funding and the discovery of the etiological DMD gene mutation, which shifted research focus toward gene-targeted therapeutic development. Nevertheless, ASA could be a powerful therapeutic for skeletal/striated muscle wasting due to its role in driving the purine nucleotide cycle (PNC) [5,6,7]. Within skeletal muscle, the PNC regulates energy balance during extreme metabolic demand (e.g., high-intensity exercise, growth); purine and pyrimidine biosynthesis necessary for DNA, RNA and enzyme cofactor production (which ultimately enables biomass accretion); and importantly, the excretion of otherwise toxic ammonia [6,8] (reviewed in [9]). There is new evidence that ASA is also capable of modulating insulin secretion and glucose homeostasis [10].

Outside of the 10-year clinical trial data indicating its safety, there are no pharmacokinetic, pharmacodynamic or toxicological data available for ASA. During the ASA trial, biofluid markers of liver (bilirubin), kidney (BUN, creatinine) and haematological (RBC, WBC, haemoglobin) function were not affected by 1–200 mg/kg daily ASA treatment [1]. The drug was administered initially via the subcutaneous route and later by intraperitoneal port when the dose escalated beyond the capacity for effective delivery via injection or insulin pump. However, the maximum dose reached was 300 mg/kg/day and higher dosages or more frequent delivery may be required to correct purine nucleotide biochemistry in disease, and especially to normalise muscle ASA levels during metabolic stress. For this reason, oral delivery may be favourable but with a potential risk to the liver during first-pass metabolism. At the conclusion of the ASA trial, bis in die oral delivery of 5 mg ASA was administered to the few remaining patients but there are no corresponding biofluid data to indicate safety at this orally administered higher dose [11].

In this study, we aimed to establish the first acute toxicology data for ASA. This data is critical to progress the clinical development of ASA since indicated safety is a necessity for regulatory approval of early-stage clinical trials [12,13,14]. In the first instance we assessed dose escalation in vitro using immortalised human myoblasts derived from a healthy paediatric patient, to assess the toxicity IC_50_ and provide application context. Specifically, we examined myoblast survival using end-point cell density as well as real-time bioimpedance monitoring. In the second part of the study, we assessed acute toxicity in vivo using Organisation for Economic Co-operation and Development (OECD) testing guidelines in mice to ascertain the LD_50_ [15]. Furthermore, our in vivo study enabled us to assess dose–response effects in the gastrointestinal, hepatic, renal and haematological systems using a combined approach of histopathology, haematology, and serum biochemistry. Herein, we provide the first evidence that ASA is a non-toxic compound following acute, high-dose exposure.

## 2. Results

**Figure 1:**
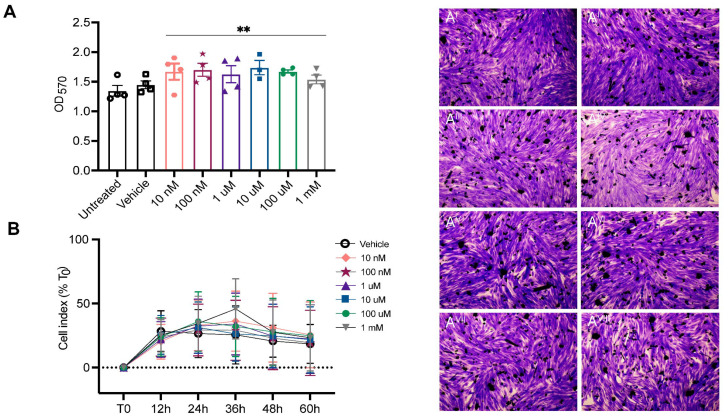
Adenylosuccinic acid (ASA) is non-toxic to human myoblasts. Across the 10 nM–1 mM dose range, ASA increased viability of myoblasts as determined via crystal violet absorbance (**A**). Representative images of crystal violet staining in untreated (**A^I^**) and vehicle (**A^II^**)-treated myoblasts and 10 nM (**A^III^**), 100 nM (**A^IV^**), 1 µM (**A^V^**), 10 µM (**A^VI^**), 100 µM (**A^VII^**) and 1 mM (**A^VIII^**) ASA-treated myoblasts. The real-time cell index was unaffected by ASA treatment across the 10 nM–1 mM range over 60 h (**B**). Treatment effect: ** *p* < 0.01.

ASA is non-toxic to human myoblasts. The effect of ASA on myoblast viability was assessed across a dose range of 10 nM–1 mM using end-point crystal violet staining and real-time Xcelligence bioimpedance monitoring. Rather than causing toxicity, the ASA treatment increased the viability of myoblasts in a non-dose-dependent manner as indicated via crystal violet absorbance (Figure 1A). The cell index was monitored in real time for 60 h post-treatment to assess the effect of the ASA treatment across the same 10 nM–1 mM range on myoblast proliferation rate. There was no effect of ASA at any dose on the real-time cell index over 60 h (Figure 1B). Our data demonstrate that ASA is non-toxic when applied directly to cells up to a concentration of 1 mM, highlighting that the cytotoxic IC_50_ is >1 mM.

ASA is non-toxic to healthy mice. Mice were dosed once with ASA dissolved in milli Q at a concentration of either 175, 550, 1750 or 5000 mg/kg (Table 1). There were no mortalities observed with ASA treatment at any dose. Since all mice survived the upper limit test (i.e., 5000 mg/kg ASA), a further two mice were administered 5000 mg/kg ASA and survived. The LD_50_ of ASA was determined to be >5000 mg/kg consistent with a non-toxic compound. 

**Figure 2:**
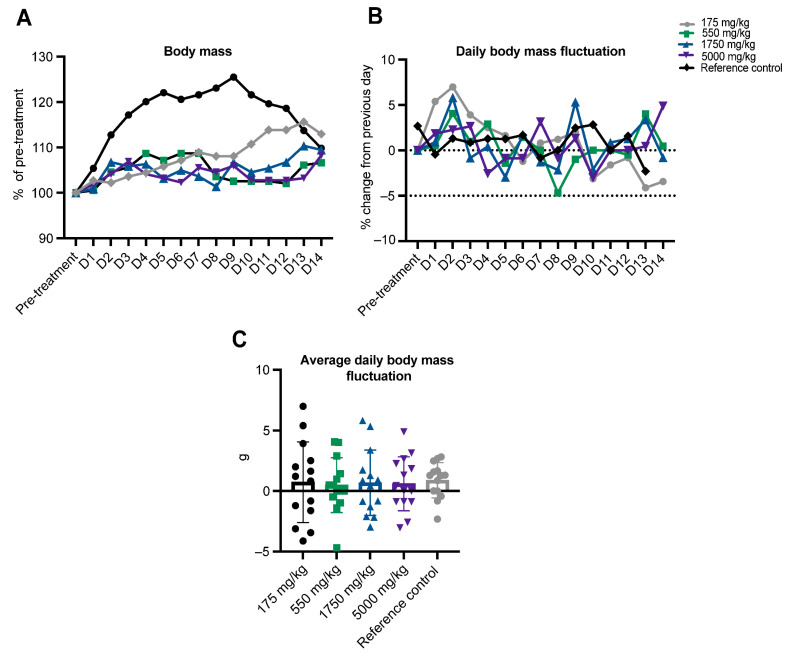
Adenylosuccinic acid (ASA) does not affect mouse body weight. Body weight was measured pre-treatment and up to 14 days post-treatment to observe if ASA was detrimental to body mass regulation (as indicated by 5% loss of body weight). Across the post-treatment period, no mouse lost >5% body weight following treatment with any ASA dose (175–5000 mg/kg; (**A**–**C**)).

Body mass. Mice were weighed just prior to treatment (following a 4 h fasting period) and daily for 14 days after ASA administration. A 5% loss of body mass represents a toxicologically meaningful observation. No mouse fell below the pre-treatment body mass on any day of the 14-day observation period (Figure 2A). Day-to-day body mass changes were also plotted. While all mice (including the untreated control) lost body mass or failed to gain body mass over at least one 24 h period during the clinical observation period (Figure 2B), no mouse lost >5% from the previous day in any one 24 h period (Figure 2B,C).

Open field observations. Mice were observed for 14 days for changes in food and water consumption, as well as symptoms of toxicity in the neuromotor, respiratory, gastrointestinal, integumentary and pain systems (summarised in Table 2). There were no adverse symptoms observed at any dose below 5000 mg/kg. However, increased motor activity was observed in the first 0.25 h and transient diarrhea was observed from 8–12 h following the 5000 mg/kg ASA treatment.

Haematology and serum biochemistry. On post-treatment day 14, blood was removed from live mice via tail venipuncture and tested for biochemical and haematological abnormalities. The data are summarised in Table 3 relative to published reference ranges for female C57Bl/6J mice [16] and an untreated C57Bl/10 control. There was no specific effect of ASA dose escalation on the haematological parameters. Segmented neutrophil concentration was higher than the reference range in the mice treated with 550 and 5000 mg/kg ASA but it was also higher in the untreated reference control suggesting this was not an effect of ASA per se. Lymphocyte concentration was higher than the reference range in the mouse treated with 175 mg/kg but was either normal or lower than the reference range in mice treated with ASA at all other doses. Eosinophil concentration was 7 times higher than the upper reference value in the mouse treated with 5000 mg/kg ASA, which may represent a drug-related effect. 

There was no ASA treatment-specific effect on serum biochemistry. All mice tested had relatively low serum total protein (mice treated with 550 mg/kg and 5000 mg/kg ASA fell below the normal reference range for this parameter) and a 3-fold lower urea concentration than the reference range. In contrast, all mice for which serum glucose concentration was available (e.g., mice treated with 175 mg/kg, 550 mg/kg and 1750 mg/kg ASA) showed values above the reference range. These data appear related to metabolism of the C57Bl/10 strain and/or the 4 h fast prior to ASA dosing that is stipulated by the testing guidelines, rather than an effect of ASA treatment per se. Mild changes to serum electrolytes were also observed. However, again, this effect was not treatment-specific and was discerned an artefact of cardiocentesis by the veterinary pathologist. The liver function enzyme, ALT, was lower than the reference for the 175 mg/kg and the 5000 mg/kg ASA mice and was at the lowest end of the reference range for the untreated reference mouse (42 U/L). However, ALT was above the reference range for the 1750 mg/kg ASA mouse, which also had a ~2-fold higher serum AST concentration indicative of liver damage. Since 5000 mg/kg ASA did not cause the same, or higher ALT and AST levels, this effect appears to be specific to the mouse rather than the escalating ASA dosage. There was no effect of ASA at any dose on serum ALP or cholesterol concentrations.

Macropathology. Mice were humanely killed and underwent a full necropsy. Splenic melanosis—a relatively common observation in mice—was observed in mice treated with 550 and1750 mg/kg ASA (Table 4) and was incidental. There were no gross macropathologic changes observed in any other organ harvested from these mice or in any organ from the 175 and 5000 mg/kg ASA mice or the reference control mouse (Table 4).

**Figure 3:**
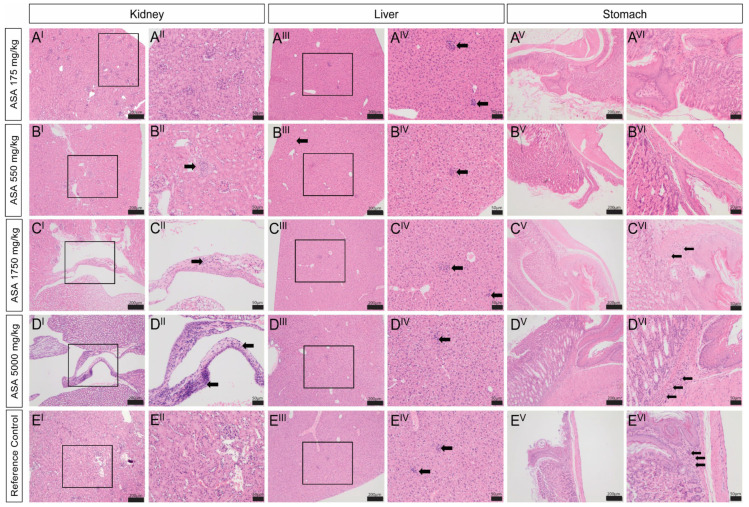
Effect of adenylosuccinic acid (ASA) on kidney, liver, and stomach histopathology. In the kidney (**A^I^**^,**II**^–**E^I^**^,**II**^), sporadic areas of mild focal interstitial lymphoplasmacytic infiltrate with fibrosis were observed (**B^II^**,**C^II^**) but were not dependent on ASA. Areas of focal necrosis with neutrophil infiltrate were also observed in the liver (**A^III^**^,**IV**^–**E^III^**^,**IV**^) and were more frequent in the mouse treated with 5000 mg/kg ASA (**D^IV^**). In the stomach (**A^V^**^,**VI**^–**E^V^**^,**VI**^), low-grade focal granulocytic gastritis was evident in the stomachs of mice treated with 1750 mg/kg ASA (**C^VI^**) and 5000 mg/kg ASA (**D^VI^**), and the untreated reference control (**E^VI^**). Images in (**A^II^**^,**IV**,**VI**^–**E^II^**^,**IV**,**VI**^) are callouts from images in (**A^I^**^,**III**,**V**^–**E^I^**^,**III**,**V**^), respectively, as indicated by the black boxes. Images in (**A^I^**^,**III**,**V**^–**E^I^**^,**III**,**V**^) are ×4 magnification (scale bar = 50 μm) and in (**A^II^**^,**IV**,**VI**^–**E^II^**^,**IV**,**VI**^) are ×10 magnification (scale bar = 200 μm). Black arrows indicate histopathology.

Histopathology. Rare lymphoplasmacytic infiltrate with mild fibrosis was observed in the kidneys of mice treated with 550–5000 mg/kg ASA (1+) but not in the kidneys of the mouse treated with 175 mg/kg ASA or the untreated mouse (Figure 3(A^I,II^–E^I,II^) and Table 4). In the liver, multiple random foci of hepatocellular necrosis with neutrophil aggregates were observed in all mice to differing extents (Figure 3(A^III,IV^–E^III,IV^) and Table 4) and was not associated with ASA treatment. In contrast, mild hepatocellular anisocytosis with scant megalocytosis was observed only in the ASA treated mice and escalated in severity in the mouse treated with 5000 mg/kg ASA (Figure 3(A^III,IV^–D^III,IV^) and Table 4). The isolated interstitial lymphoplasmacytic infiltrate of the kidney, and focal necrosis and mild anisocytosis of the liver are common background findings in laboratory mice [20]. 

No significant abnormalities were observed in the stomach of the mice treated with 175 mg/kg or 550 mg/kg ASA. However, mild to moderate focal neutrophilic infiltrate into the gastric mucosa at the glandular and non-glandular margin and mild focal granulocytic gastritis was observed in the stomach of the untreated mouse and the mice treated with 1750 and 5000 mg/kg ASA. Gastritis and neutrophilic infiltrate in the gastric mucosa are notable background findings in laboratory mice (personal observations, L. Rasmussen) as indicated by the fact that the intensity of the pathology was equivalent between the mouse treated with 5000 mg/kg ASA and the untreated reference control. 

## 3. Discussion

The first part of our study revealed ASA as a non-toxic compound to human myoblasts in vitro. ASA was non-toxic to cells at any dose within a 10 nM–1 mM range and therefore has a cytotoxic IC_50_ > 1 mM. In fact, in the end-point cell density assay, ASA (any dose) treatment for 4 days resulted in a higher number of live myoblasts than in the untreated or vehicle-treated control cultures. Purine nucleotides are the fundamental building blocks of molecules that regulate cell life, including DNA, RNA, enzyme cofactors and high-energy phosphates (ATP, ADP, AMP) [2]. They also function in the signal transduction of critical cell survival responses [10,21,22,23,24]. ASA appears to support cell survival in vitro, but does not impact the rate of myoblast proliferation per se. 

The second part of our study revealed that ASA was non-toxic to mice in vivo at the upper acute toxicity testing limit of 5000 mg/kg and caused no adverse clinical symptoms in the open field. For this reason, a limit test was applied at 5000 mg/kg. Although drug testing in the GHS Category 5 range (2000–500 mg/kg) is usually discouraged within the acute toxicity test, in the previous ASA clinical trial, dose escalation was required to lower the clinical biomarkers of muscle degradation as patients attained muscle biomass during adolescence and the disease progressed [1]. High ASA doses will likely be needed to biochemically correct primary and secondary PNC deficits in the clinical setting. Three mice survived the limit test and only mild, sporadic symptoms were observed. For example, in the 0.25 h immediately after treatment, 5000 mg/kg ASA induced hyperactivity in the open field and transient diarrhea that resolved within the first 24 h period. These data suggest gastrointestinal discomfort could be symptomatic of acute, high-dose ASA treatment. Weight loss was observed at day 12 of the 14-day observational period and may be related or unrelated to ASA exposure. While there were no macro- or histo-pathological changes observed in the gastrointestinal tract due to ASA exposure, other similar metabolite small molecules are associated with gastrointestinal symptoms. ASA generates fumarate as a by-product of its enzymatic degradation by adenylosuccinate lyase (ADSL) within the PNC [8]. Synthetic fumarate drugs used in the clinical management of multiple sclerosis (MS) and psoriasis, such as dimethyl- and monomethyl-fumarate [25], are known to aggravate the gastrointestinal tract resulting in diarrhea, nausea and stomach cramps and pain in up to 60% of patients [26,27,28]. At least in the context of MS, gastrointestinal complaints are usually mild in severity and resolve within the first 2 months of treatment [29,30,31]. However, they are the most frequent cause of treatment discontinuation [29]. If a proportion of ASA metabolism occurs in the gut, either within the gastric tissues or resident microbiome, endogenous fumarate production could be responsible for similar symptoms and ultimately manifest as weight loss. Our findings suggest sub-/chronic studies should be pursued to understand whether ASA shares a similar side-effect profile to fumarate compounds, and whether conventional clinical management strategies, such as dosing with food, bis in die, or formulated in slow-release packaging could minimise gut symptoms.

There were some haematological anomalies observed in the ASA-treated mice that were deemed to be an artefact of cardiocentesis—particularly the blood electrolyte concentration. Unfortunately, there was an insufficient blood sample to detect blood electrolytes in the untreated reference control for comparison. Sodium and chloride concentration were both lower than the reference range in blood collected from the mice with a sufficient sample available (the 175 and 1750 mg/kg ASA-treated mice). Although the mechanism of ASA transport across cell membranes has not been established, we previously hypothesised that a likely transport mechanism is via the transmembrane solute carrier (SLC) as per fumarate and citrate [9]. Di- and tri-carboxylate SLCs are sodium-dependent symporters while the glycine SLC is a sodium/chloride symporter [32]. If ASA is transported via SLCs its administration could be associated with fluctuations in the sodium and/or chloride concentration of the extracellular and intracellular compartments. No changes in blood electrolyte levels were reported in the ASA clinical trial throughout its duration [1]. However, ASA was administered in the trial as a tetra-sodium salt formulation [1]. Sodium-based salt formulations are ideal for increasing the absorption of weak acids across the GI tract and their bioavailability to target tissues [33]. In future trials, the formulation may need to consider ASA’s uptake route and the potential requirement of electrolytes to support maximal absorption.

Adverse drug reactions are a frequent and challenging aspect of modern medicine [34]. Ultimately, a drug’s benefit must be weighed against its side-effect profile and provide a significant advantage to human livelihood. Oral administration has benefits over injectable delivery via the subcutaneous, intraperitoneal and intravenous routes, in that large quantities of drug can be sustainably delivered with minimal medical intervention [33]. However, a significant drawback is that the liver is subjected to the highest drug concentration during first-pass metabolism following drug absorption across the gastrointestinal tract. As a result, drug toxicity is commonly observed in the liver (second only to the cardiovascular system as a site of toxicology attrition [35]) as hepatocellular injury and the release of enzymes, e.g., ALT, AST and ALP, into the blood circulation. In our study, it was difficult to discern random incidental histopathologic events from those caused specifically by ASA. Background lesions that are commonly observed in mice with no significance are splenic melanosis, interstitial lymphoplasmacytic infiltrates in the kidney, random foci of hepatocellular necrosis with neutrophil aggregates and hepatocellular anisocytosis with megalocytosis [20]. Granulocytic gastritis is also frequently observed (Personal observations, L. Rasmussen). All of these pathologies were seen to some extent in our mice and appeared independent of ASA treatment. However, it cannot be ruled out that moderate- to high-dose ASA may cause focal injury to both the liver and kidney. While dose escalation did not intensify kidney histopathology, high (5000 mg/kg)-dose ASA increased the severity of histopathologic symptoms in the liver. Since the bioavailability and tissue distribution of oral ASA treatment is currently unknown, it should be assumed that high-dose delivery could be needed to normalise dysregulated purine biochemistry in relevant diseases and required on a chronic basis [9]. As such, our histopathology data warrant follow-up with sub-/chronic studies testing low–high dosages to establish longitudinal safety.

Idiosyncratic drug toxicities are generally unpredictable and can present as delayed onset. Thus, these toxicities are typically not revealed during acute toxicity testing. Their proposed mechanisms include via metabolic, haptenogenic, inflammogenic and/or danger signalling, or via reversible drug induction of the immunological response [35]. Our previous work investigating ASA’s mechanism(s) of action demonstrates regulation of high-energy phosphate metabolism (e.g., ATP and the phosphocreatine pool) [36], suppression of inflammation and the immune systems similar to other dicarboxylate metabolites (e.g., β-hydroxybutyrate) [9], and activation of the danger signalling transcription factor, nuclear factor-erythroid factor 2-related factor 2 (Nrf2) [21]. These mechanisms of action could render ASA particularly prone to isolated toxicity due to nuances in inflammation and immune system function between individuals. A relatively rare complication of dimethyl fumarate treatment in the context of MS is lymphopenia (<2% of consumers) which is more frequent in older males [37,38]. Purportedly, lymphopenia persists in many of these patients after treatment discontinuation demonstrating that fumarate induces long-term changes to the immune system and that person-specific autoimmune factors may be involved [31]. High-dose ASA (1750 and 5000 mg/kg) was associated with lymphopenia in our haematological observations and should be followed up in sub-/chronic toxicology studies. Ultimately, detailed toxicity surveillance within the clinical trial setting will be required, especially over the long term, to determine if ASA treatment is safe for all individuals irrespective of underlying conditions. 

## 4. Materials and Methods

### 4.1. In Vitro Toxicity Testing

The potential cytotoxicity of ASA was assessed in in vitro cell culture. Immortalised skeletal muscle myoblasts were cultured in growth medium, seeded at a density of 90–100,000 cells per well and passaged every 3 days [36,39]. The maximal tolerable dose of ASA was assessed using two methods: end-point crystal violet staining was used to quantitate myoblast viability while an xCELLigence bioimpedance system was used to quantitate real-time myoblast cell index. For the crystal violet assay, myoblasts were grown in media only, or media supplemented with vehicle (milliQ H_2_O) or increasing concentrations of ASA (10 nM–1 mM) in vehicle. Following 4 days incubation, cells were stained with 0.1% crystal violet solution and absorbance was measured spectrophotometrically at 570 nm [40]. For cell proliferation assay, myoblasts were seeded at 5000 cells/well and once adhered, were treated with 50 μL media, media with vehicle or media with increasing concentrations of ASA (10 nM–1 mM). Cell proliferation was monitored in real-time using an xCELLigence RTCA MP system in which the cell index was measured every 30 min for 4 days.

### 4.2. In Vivo Toxicity Testing

#### 4.2.1. Animals

Animal experimentation was approved by the Victoria University Animal Ethics Committee (17/006) and performed in accordance with the Australian Code of Practice for the Care and Use of Animals for Research Purposes. Female C57Bl/10 mice aged 8 weeks were used in the in vivo studies and purchased from the Animal Resource Centre (Murdoch, WA, Australia). Mice acclimatized for one week prior to testing in the Victoria University (Western Centre for Health Research and Education, Sunshine Hospital) vivarium. Animals were maintained on a 12:12 light: dark cycle at 23 °C (21% humidity) with unrestricted access to standard rodent chow and water.

#### 4.2.2. Drug Details

ASA (CAS 19046-78-7; C_14_H_18_N_5_O_11_P; 463.29 m.w.; 95% purity) was purchased from Santa Cruz (sc-214511; Dallas, TX, USA). Doses were prepared in double-distilled milliQ H_2_O relative to body weight as described in Table 1.

#### 4.2.3. Acute Oral Toxicity Procedure

The OECD “Up-and-down” toxicity test was implemented to evaluate ASA [15]. Mice (*n* = 1/dose) were sequentially dosed with increasing concentrations of ASA dissolved in milliQ water using an incremental scale of 3.2 (i.e., 175, 550, 1750 mg/kg in the first instance). The commencing dose of 175 mg/kg was selected since lower doses (corrected for the different metabolic rates of mice compared to humans) were demonstrably safe in humans in the ASA clinical trial [1]. The dose was only increased when no signs of toxicity were observed in the first 48 h. In-life observations included monitoring of body weight, motor activity, neurological, respiratory and gastrointestinal function, skin and mucous membrane changes, and pain. Mice were observed for 14 days post-treatment. The first mouse was dosed with 175 mg/kg ASA via oral gavage (21G) and monitored for 48 h. Since no signs of toxicity were observed, a second mouse was dosed with 550 mg/kg ASA and monitored for 48 h. This was repeated for the 1750 mg/kg dose. The upper limit dosage for the up-and-down procedure is 2000 mg/kg, but since no signs of toxicity were observed at the relatively equivalent dose of 1750 mg/kg we added an upper limit test of 5000 mg/kg to the protocol as recommended by the OECD test. At the conclusion of the observation period, mice were transported to an independent commercial laboratory animal testing facility (Cerberus Sciences, Scoresby, VIC, Australia). 

#### 4.2.4. Haematology

Blood was removed from anaesthetised mice via cardiocentesis into EDTA-coated vacutainer tubes. Blood samples were transferred to a commercial veterinary pathology testing laboratory (ASAP Laboratories, Mulgrave, VIC, Australia) and tested for haematocrit, haemoglobin, total erythrocyte, platelet, leukocyte, neutrophil, lymphocyte, monocyte, eosinophil, and basophil count using a Siemens ADVIA 2120^®^ haematology system. 

#### 4.2.5. Biochemistry

Whole blood was centrifuged at 3000× *g* for 10 min at 4 °C. Serum was separated and analysed for routine chemistry including glucose, creatinine, urea, aspartate aminotransferase, alanine aminotransferase, alkaline phosphatase, total cholesterol, triglycerides, uric acid, albumin, and total proteins using a Beckman Coulter (Brea, CA, USA) AU680 chemistry system.

#### 4.2.6. Histopathology

Mice were killed via CO_2_ asphyxiation and a full necropsy was performed. Liver, kidney, and stomach were further processed for histopathological analysis. Samples were fixed in 10% neutral buffered formalin, sectioned into histology cassettes, and processed through a haematoxylin and eosin stain. Photomicrographs were taken using an Olympus BX41 microscope and LC30 camera and software. Histological photomicrographs were graded by an independent, certified specialist veterinary pathologist (Dr Lorna Rasmussen, B. VSc., B. VSc. (Hons), M. MedVet (Path)., Dip. ACVP). An untreated, sex- and age-matched control mouse was included in the macro- and micro-pathology analysis as a phenotype and environmental control to validate reference values species/strain-related nuances.

## 5. Conclusions

Our acute toxicity study revealed ASA as a non-toxic compound (LD_50_ > 5000 mg/kg) without obvious impact on macropathology following oral administration. There were incidental non-dose-dependent lesions noted in the liver, kidney and gastrointestinal tract common to the *mus musculis* species [20]. However, it cannot be ruled out that the ASA treatment was involved, especially at higher dosages where all these organs were subjected to high concentrations of the metabolite (gut and liver) or its excretion by-product (urea). These observations require follow up sub-chronic and chronic toxicology studies to confirm that ASA is non-toxic over the long term. 

## Figures and Tables

**Table 1:** Dose preparation of adenylosuccinic acid (ASA).

Dose	Post-Fasted Body Weight (g)	ASA Mass (mg)	Volume H_2_O/Dose (mL)
175 mg/kg	20.4	3.57	0.204
550 mg/kg	19.6	10.78	0.196
1750 mg/kg	22.2	38.85	0.222
5000 mg/kg	21.8	109	0.218

**Table 2:** Effects of acute adenylosuccinic acid (ASA) exposure (175–5000 mg/kg) on in-life observations. Key: - = no changes, + = mild changes.

		Observations				
Observations	Symptoms	175 mg/kg ASA *n* = 1	550 mg/kg ASA *n* = 1	1750 mg/kg ASA *n* = 1	5000 mg/kg ASA *n* = 1	Untreated
Body weight	>5% loss of body weight	-	-	-	-	-
Food and water consumption	-	-	-	-	-	-
Motor activity	Home-cage activity	-	-	-	+ (<30 m)	-
Neurological system	Tremors, limb tone, ataxia	-	-	-	-	-
Respiratory system	Gasping, heaving, cyanosis	-	-	-	-	-
Gastrointestinal function	Abdominal griping, vomiting, diarrhoea	-	-	-	+ (diarrhoea)	-
Skin and mucous membranes	Secretions, excretions	-	-	-	-	-
Pain	Grimacing, altered social activity	-	-		-	-

**Table 3:** Effects of acute adenylosuccinic acid (ASA) exposure (175–5000 mg/kg) on serum biochemistry. Key: * = outside of reference range; ^a^ [17]; ^b^ [18]; ^c^ [17]; ^d^ [19]; IS = insufficient sample to measure analyte; MCH = mean corpuscular haemoglobin; MCHC = mean corpuscular haemoglobin concentration; MCV = mean corpuscular volume; WCC = white cell count.

Parameters	Reference Range	ASA Dose				Reference Control
		175 mg/kg	550 mg/kg	1750 mg/kg	5000 mg/kg	
Haematological						
Erythrocytes (×10^12^/L)	3.47–11.73 ^a^	10.5	11.06	10.66	11.0	11.02
Haematocrit (L/L)	0.16–0.58 ^a^	0.48	0.54	0.54	0.54	0.54
Haemoglobin (g/L)	57.0–170.0 ^a^	156	162	153	157	158
MCV (fL)	25.7–59.7 ^b^	48	49	50	49	49
MCH (pg)	9.0–19.2 ^b^	16	15	14	14	14
MCHC (g/L)	264.6–314.2 ^b^	323 *	300	286	289	290
Platelet (×10^9^/L)	520–1880 ^b^	942	954	1040	1077	1188
WCC (×10^9^/L)	2.2–11.53 ^a^	8.9	5.5	3.8	4.6	5.8
Segmented Neutrophils (×10^9^/L)	0.12–0.30 ^b^	0.3	0.7 *	0.2	0.4 *	0.5 *
Lymphocytes (×10^9^/L)	4.36–5.68 ^b^	8.5 *	4.6	3.4 *	3.4 *	4.8
Monocytes (×10^9^/L)	0–0.3 ^b^	0.18	0.06	0.23	0	0
Eosinophils (×10^9^/L)	0–5.1 ^d^	0	2	0	7*	0.1
Basophils (×10^9^/L)	0–0.1 ^d^	0	0	0	0.1	0
Biochemical						
Total Protein (g/L)	45–83 ^a^	48	42 *	53	40 *	53
Albumin (g/L)	20–47 ^a^	29	24	30	21	29
Total globulin (g/L)	18–21 ^c^	19	18	23 *	19	24
Glucose (mmol/L)	5.2–12.2 ^a^	18.1 *	13.8 *	15 *	IS	IS
Sodium (mmol/L)	149.0–281.4 ^a^	142 *	IS	145 *	IS	IS
Potassium (mmol/L)	4.0–14.0 ^a^	10.8	IS	7.5	IS	IS
Chloride (mmol/L)	110.0–204.4 ^a^	98 *	IS	101 *	IS	IS
Calcium (mmol/L)	2.3–3.5 ^a^	2.64	2.15 *	2.57	1.94 *	2.66
Phosphorous (mmol/L)	2.0–3.1 ^a^	3.32 *	2.23	3.95 *	2.83	3.69
Urea (mmol/L)	14.5–21.4 ^a^	4.1 *	5.3 *	5.5 *	4.9 *	5.4 *
Total bilirubin (µmol/L)	3.4–14.3 ^a^	9	1 *	4	IS	IS
Alanine aminotransferase (ALT; U/L)	42–73 ^a^	39 *	50	75 *	25 *	42
Aspartate aminotransferase (AST; U/L)	51–122 ^a^	97	76	220 *	62	86
Alkaline phosphatase (ALP; U/L)	103–217 ^a^	140	155	193	137	178
GammaGT (U/L)	6.0–8.0 ^a^	7	<5 *	<5 *	IS	IS
Creatine kinase (U/L)	105–649 ^a^	100 *	43 *	290	IS	IS
Total cholesterol (mmol/L)	1.3–3.4 ^a^	2.02	1.77	2.29	2.02	2.27

**Table 4:** Effects of acute adenylosuccinic acid (ASA) exposure (175–5000 mg/kg) on macro- and histo-pathology. Key: - = no changes, + = mild changes, ++ = moderate changes, +++ = significant changes.

Pathology	Symptoms	ASA Dose				
		175 mg/kg	550 mg/kg	1750 mg/kg	5000 mg/kg	Reference Control
Macropathology	Splenic melanosis (background lesion)	-	+	+	-	-
Kidney	Rare interstitial lymphoplasmacytic infiltrate with fibrosis	-	+	+	+	-
Liver	Multiple random foci of necrosis with neutrophil aggregates	+++	+++	+	+	+++
	Mild anisocytosis with scant megalocytosis	+	+	+	++	-
Stomach	Focal neutrophilic infiltrate into gastric mucosa;	-	-	+	++	+
	Low-grade focal granulocytic gastritis	-	-	+	+++	+++

## Data Availability

Data will be made available upon request.
